# Outcomes with Single Tremelimumab Regular Interval Durvalumab (STRIDE) for Unresectable Hepatocellular Carcinoma in the US Veterans Administration

**DOI:** 10.3390/cancers18071085

**Published:** 2026-03-26

**Authors:** Shalini Bansal, Priya Amin, Courtney Williamson, Stephen J. Valerio, David E. Kaplan

**Affiliations:** 1 Gastroenterology Section, Corporal Michael J. Crescenz VA Medical Center, Philadelphia, PA 19104, USA; 2 School of Medicine, Thomas Jefferson University, Philadelphia, PA 19107, USA; 3 AstraZeneca, Gaithersburg, MD 20878, USAstephen.valerio@astrazeneca.com (S.J.V.); 4 Department of Medicine, Perelman School of Medicine, University of Pennsylvania, Philadelphia, PA 19104, USA

**Keywords:** carcinoma, hepatocellular, safety, survival, therapeutics

## Abstract

**Background:** HIMALAYA demonstrated that STRIDE (Single Tremelimumab Regular Interval Durvalumab) significantly improved overall survival (OS) compared with sorafenib in participants with unresectable hepatocellular carcinoma (HCC). This retrospective, real-world cohort study evaluated outcomes with STRIDE in veterans with HCC. **Methods:** Patients diagnosed with HCC between 1 January 2008 and 28 February 2024 who received ≥1 dose of STRIDE for unresectable disease were included. Data were collected from the Veteran Affairs Corporate Data Warehouse. Safety and efficacy were evaluated overall and for subgroups of patients with Child–Pugh A versus Child–Pugh B cirrhosis, viral versus non-viral HCC, and those with versus without prior non-systemic therapies. **Results:** Overall, 107 patients (100.0% male) were included. Median (interquartile range) age was 72.2 (68.0–76.1) years. There were 22 Grade 3–4 adverse events reported (three in patients with Child–Pugh B cirrhosis). Median OS (95% CI) was 12.4 (9.1–22.1) months and 5.2 (1.5–9.3) months in patients with Child–Pugh A (*n* = 81; 75.7%) and Child–Pugh B cirrhosis (*n* = 26; 24.3%), respectively. In patients with viral (*n* = 64; 59.8%) versus non-viral etiology (*n* = 43; 40.2%), median OS (95% CI) was 10.5 (7.0–25.6) months versus 9.0 (4.6–16.0) months, respectively. In patients without (*n* = 30; 28.0%) versus with prior non-systemic therapies (*n* = 77; 72.0%), median OS (95% CI) was 7.7 (2.8–17.3) months versus 11.1 (7.6–17.6) months, respectively. **Conclusions:** These results suggest that STRIDE is well tolerated and may offer a survival benefit to a broad range of patients with unresectable HCC, representing populations that are more reflective of real-world clinical practice.

## Simple Summary

STRIDE (Single Tremelimumab Regular Interval Durvalumab) has been shown to be a safe and effective treatment option for people with unresectable hepatocellular carcinoma (HCC). However, there is limited published information on real-world outcomes. This analysis of patients with HCC in the US Veteran Affairs healthcare system showed that STRIDE was a safe option, regardless of whether or not patients received prior non-systemic therapies. Similarly, viral versus non-viral etiology did not lead to a detectable impact on outcomes. Participants with Child–Pugh B liver function excluded from the HIMALAYA study were also safely treated with STRIDE, with no increase in safety events compared with participants with Child–Pugh A liver function.

## 1. Introduction

Hepatocellular carcinoma (HCC) represents a significant global health burden, ranking as the third leading cause of cancer-related mortality worldwide, with approximately 758,000 liver cancer-related deaths reported annually [1]. Unresectable HCC (uHCC) poses a particular challenge, as approximately 60% of people present with advanced disease at diagnosis, significantly limiting curative treatment options and resulting in a median survival of only 6–8 months without systemic therapy [2].

Initially, multikinase inhibitors such as sorafenib and lenvatinib were the only systemic therapies approved for the first-line treatment of uHCC [3]. In recent years, several phase 3 clinical studies have demonstrated the efficacy of immune checkpoint inhibitor (ICI)-based combination therapies as first-line treatments in this population [4,5,6,7]. In the phase 3 HIMALAYA study (NCT03298451), the STRIDE (Single Tremelimumab Regular Interval Durvalumab) regimen significantly improved overall survival (OS) versus sorafenib in uHCC [4]. STRIDE is the only dual-ICI regimen that has shown 5-year OS rate improvement versus sorafenib (19.6% vs. 9.4%, respectively) [8].

Clinical trials typically enroll a specifically selected set of patients in a tightly regulated setting, leading to a potential gap between clinical trial efficacy and real-world outcomes, where patient populations are more diverse and healthcare settings can vary widely.

Several questions regarding responses to therapy in specific subgroups of patients remain open either due to stringent registrational trial designs, results of prespecified stratification analyses, or post-hoc subgroup analysis results. While most (97.9%) participants in the HIMALAYA study had Child–Pugh A liver function, approximately 40% of patients with uHCC present with Child–Pugh B liver function in the real-world clinical setting [4,9], yet there are limited data on the safety, tolerability, and efficacy of STRIDE in patients with Child–Pugh B liver function. Additionally, there are conflicting data regarding disease etiology (viral vs. non-viral) and outcomes with the use of immunotherapy [10]. For example, while atezolizumab plus bevacizumab appeared to demonstrate greater relative improvement in OS among participants with viral etiology in the IMbrave 150 study [11], in the HIMALAYA study, STRIDE demonstrated a clinically meaningful survival benefit regardless of viral or non-viral etiology [12]. Finally, receipt of prior non-systemic therapies may impair liver function in some patients and reduce the benefit of systemic therapy [13,14], yet there are few data assessing the effects of prior non-systemic therapies or resection on outcomes in patients who receive STRIDE. Studies employing real-world data are important for understanding the efficacy of these treatments in different healthcare settings and in broader patient populations.

The Veterans Affairs (VA) system is the largest integrated healthcare system in the US [15]. While hundreds of patients in the VA healthcare system are diagnosed with HCC each year, few are eligible for clinical trials due to compromised liver function or other disqualifying factors [16]. For this reason, it is important to understand the patient characteristics and associated outcomes in this population.

In this retrospective cohort study, we utilized data from the VA Corporate Data Warehouse (CDW) to evaluate survival outcomes with STRIDE in a real-world cohort of patients with uHCC, including patients with Child–Pugh B cirrhosis, viral versus non-viral etiologies of HCC, or prior receipt of non-systemic therapies (locoregional therapy [LRT] or resection) for uHCC. Additionally, we assess safety, patient characteristics, and factors influencing therapeutic outcomes.

## 2. Materials and Methods

### 2.1. Data Source and Ethics

Data were collected from the CDW. Data from the CDW were validated from electronic medical records (EMRs). All data were stored on a secure VA server. The study was approved as an exempt protocol (#01802) by the Institutional Review Board of the Michael J. Crescenz VA Medical Center and conducted under a waiver of requirement for informed consent.

### 2.2. Study Population

The study population included all patients with newly diagnosed HCC from 1 January 2008 to 28 February 2024 who had received ≥1 dose of first-line systemic therapy for uHCC through the VA healthcare system. Patients with prior diagnoses of cancer (except non-melanoma skin cancer and indolent genitourinary cancers) with metastatic disease to the liver were excluded. Data were collected through 30 April 2025.

Case identification was obtained from two data sources. First, the CDW was queried for International Classification of Diseases, Tenth Revision codes for HCC (C22.0 or C22.8). Pharmacy records were used to identify patients who were prescribed any systemic therapy approved by the US Food and Drug Administration for the treatment of HCC (sorafenib, lenvatinib, regorafenib, cabozantinib, ramucirumab, nivolumab, pembrolizumab, atezolizumab, or bevacizumab) at the time of study conduct. Second, 100% of patients who were exposed to STRIDE were confirmed to have HCC and exposure to at least one cycle of the STRIDE regimen via manual abstraction. Only patients confirmed to have STRIDE as the first-line systemic therapy were included. Post-abstraction exclusions were made if records did not qualify for inclusion. Missing data were excluded from summary statistic calculations.

### 2.3. Covariates

Demographics, disease characteristics, and treatment information were extracted from the CDW. Electronic Child–Turcotte–Pugh (eCTP) scores were determined at the time of initiation of STRIDE using the methods described by Kaplan et al. [17]. Eastern Cooperative Oncology Group performance status (ECOG PS), Barcelona Clinic Liver Cancer (BCLC) staging, and prior treatments were manually abstracted by a single chart reviewer using Research Electronic Data Capture (REDCap).

### 2.4. Outcomes

The primary outcome of interest was OS. OS and follow-up time were calculated from the time of initiation of STRIDE censored at the time of final extraction of death outcomes (15 April 2025). Liver function, time from diagnosis to initiation of STRIDE, number of durvalumab cycles, follow-up time, number of patients ongoing durvalumab treatment at the time of data extraction, and safety were also determined. Liver function was assessed by change in albumin–bilirubin (ALBI) score over time and time to increase in Child–Pugh score (≥2 points) in the subset of patients with Child–Pugh A versus Child–Pugh B liver function. Number of treatment cycles and adverse events (AEs) were manually abstracted by a single chart reviewer using REDCap (version 5).

### 2.5. Statistical Analyses

Demographics, disease characteristics, and outcomes were described overall and by Child–Pugh score, viral etiology, and prior receipt of non-systemic therapies (LRT or resection). The significance level for all statistical analyses was a two-sided, *p* < 0.05. All analyses were conducted using R version 4.3. Survival outcomes were evaluated using Kaplan–Meier methodologies. A sensitivity analysis of median OS was conducted within a cohort of patients that would have met criteria for enrollment in the HIMALAYA study (Child–Pugh Class A, ECOG PS 0–1, no main trunk portal vein thrombosis [Vp4], and no prior systemic treatment for uHCC).

## 3. Results

**Figure 1:**
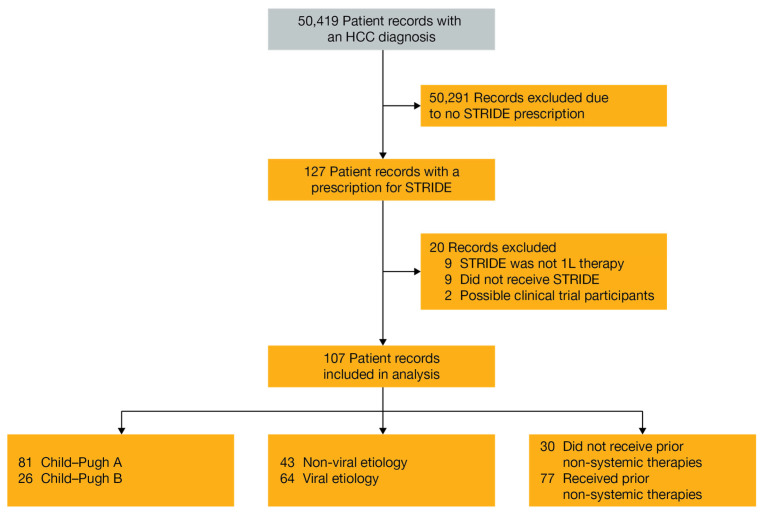
Flowchart of study cohorts. Abbreviations: 1L, first-line; HCC, hepatocellular carcinoma; STRIDE, Single Tremelimumab Regular Interval Durvalumab.

A total of 50,419 records with an HCC diagnosis were reviewed and 107 patient records were included in this analysis (Figure 1). Efficacy and safety data were available for all patient records included in the analysis. Treatment information, including time to STRIDE exposure, number of durvalumab cycles, and follow-up time are summarized in Table 1.

### 3.1. Demographics and Outcomes in Overall Population, and Sensitivity Analysis

All 107 patients were male, median (interquartile range [IQR]) age was 72.2 (68.0–76.1) years, and most patients were white (*n* = 67; 62.6%; Table 2).

**Figure 2:**
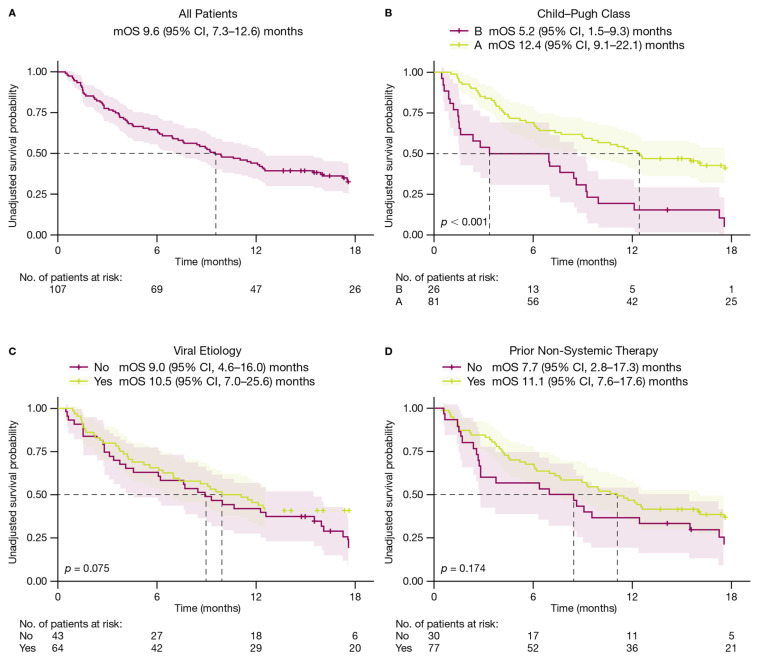
Overall survival (**A**) in all patients, (**B**) by Child–Pugh Class, (**C**) by viral etiology, and (**D**) by receipt of prior non-systemic therapy. OS was assessed by the Kaplan–Meier technique. Bootstrapping was used to estimate confidence intervals using R adjusted curves package. Dotted lines represent median event times. Abbreviations: CI, confidence interval; mOS, median overall survival.

Median OS in the broad uHCC patient population was 9.6 (95% confidence interval [CI], 7.3–12.6) months (Figure 2A). There were 19/107 (17.8%) patients with ongoing durvalumab treatment at the time of data extraction (Table 3). The most common reasons for treatment discontinuation were AEs and progressive disease (Table 3). There were 5/107 (4.7%) patients who received subsequent therapy following STRIDE (Table 4).

Overall, 74 Grade 1–2 AEs and 22 Grade 3–4 AEs were reported (Supplementary Table S1). No Grade 5 AEs were reported. The most frequent AEs in all patients were fatigue (14.6%), rash (14.6%), and diarrhea (12.5%; Supplementary Table S1).

In the sensitivity analysis there were 70 patients who met the inclusion criteria for the HIMALAYA study and 37 patients who did not. Median OS was 12.6 (95% CI, 9.1–22.1) months for patients who met the inclusion criteria for the HIMALAYA study and 7.3 (95% CI, 3.4–9.6) months for patients who did not (Supplementary Table S2).

### 3.2. Demographics and Outcomes by Child–Pugh Class

Of the 107 patients with uHCC who received STRIDE, 81 (75.7%) had Child–Pugh A liver function and 26 (24.3%) had Child–Pugh B liver function. Median age was similar for patients with Child–Pugh A liver function and for patients with Child–Pugh B liver function (72.1 [IQR, 68.5–76.4] vs. 72.9 [IQR, 66.8–76.1] years, respectively; *p* = 0.4159; Table 2).

Among the patients who had Child–Pugh A or Child–Pugh B liver function, 7/81 (8.6%) and 9/26 (34.6%) had alcoholic liver disease (ALD) etiology, 32/81 (39.5%) and 4/26 (15.4%) had hepatitis C virus (HCV) etiology, 21/81 (25.9%) and 6/26 (23.1%) had ALD plus HCV etiology, and 18/81 (22.2%) and 7/26 (26.9%) had metabolic dysfunction-associated steatotic hepatitis (MASH) cirrhosis, respectively (Table 2).

Among patients who had Child–Pugh A liver function, 24/81 (29.6%) exhibited modified ALBI Grade 1 liver function, 20/81 (24.7%) had modified ALBI Grade 2A, 33/81 (40.7%) had modified ALBI Grade 2B, and 3/81 (3.7%) had modified ALBI Grade 3 liver function at baseline. Among patients who had Child–Pugh B liver function, there were no patients with modified ALBI Grade 1 or 2A liver function, 15/26 (57.7%) had modified ALBI Grade 2B liver function, and 10/26 (38.5%) had modified ALBI Grade 3 liver function at baseline.

Extrahepatic spread was more frequent among patients who had Child–Pugh A liver function (22/81 [27.2%]) than patients who had Child–Pugh B liver function (2/26 [7.7%]; *p* = 0.07; Table 2). Macrovascular invasion was less frequent among patients who had Child–Pugh A liver function, with 2/81 (2.5%) with Vp2, 11/81 (13.6%) with Vp3, 9/81 (11.1%) with Vp4, and 3/81 (3.7%) not classified on imaging (Table 2). More than half of patients in the Child–Pugh B group had vascular invasion; of these patients, 2/26 (7.7%) had Vp3, 11/26 (42.3%) had Vp4, and 2/26 (7.7%) were not classified on imaging (Table 2).

**Figure 3:**
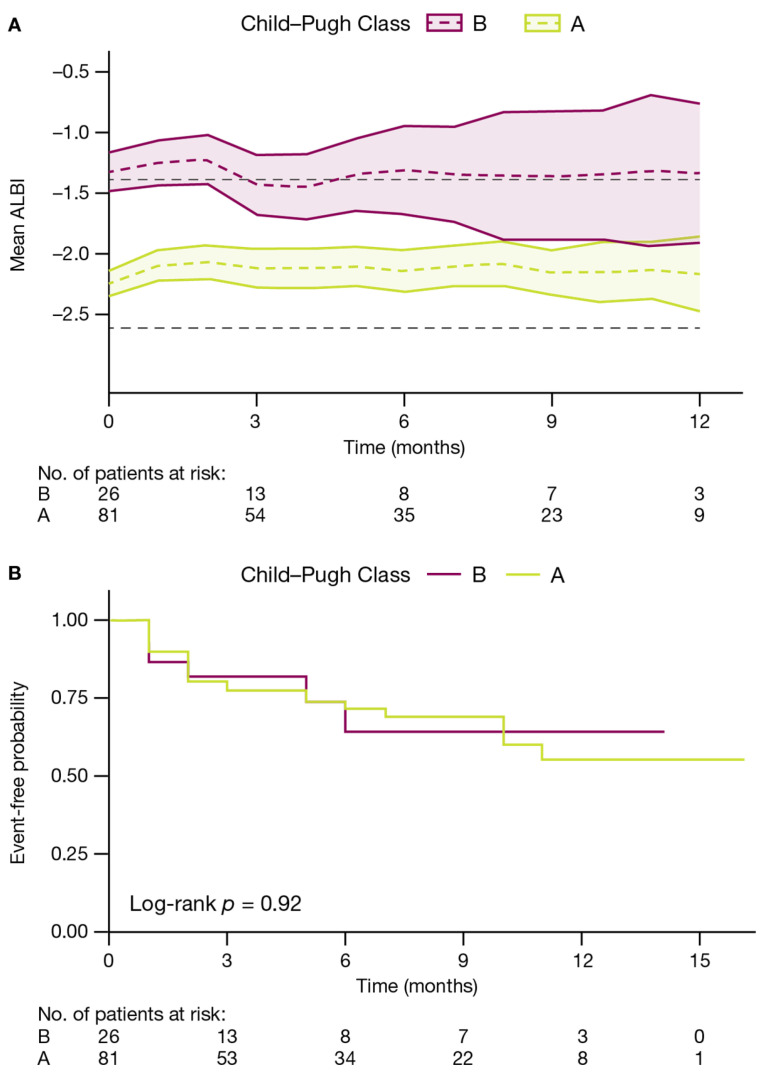
Change in liver function in patients treated with STRIDE assessed by (**A**) mean ALBI change over time and (**B**) time to increase in Child–Pugh score (≥2 points). Dashed lines reflect mean value and shaded area in Panel (**A**) represents the 95% CI. Time to increase in Child–Pugh score (Panel (**B**)) was evaluated using the Kaplan–Meier technique. Black dotted lines in Panel (**A**) reflect the cutoff points for ALBI Grade 2 and 3. Abbreviations: ALBI, albumin–bilirubin; eCTP, electronic Child–Turcotte–Pugh.

Mean ALBI score was stable during treatment with STRIDE among survivors (Figure 3A). Overall, 24/81 (29.6%) patients with Child–Pugh A liver function and 6/26 (23.1%) patients with Child–Pugh B liver function experienced a two-point worsening in Child–Pugh score. Median (95% CI) time to two-point worsening in Child–Pugh score for patients with Child–Pugh A liver function or Child–Pugh B liver function was not reached, respectively (Figure 3B).

Median OS was 12.4 (95% CI, 9.1–22.1) months in patients who had Child–Pugh A liver function and 5.2 (95% CI, 1.5–9.3) months in patients who had Child–Pugh B liver function (Figure 2B). There were 19/81 (23.5%) patients with Child–Pugh A liver function who were ongoing durvalumab treatment at the time of data extraction versus no patients with Child–Pugh B liver function (Table 3). There was a higher rate of discontinuation due to AEs in patients who had Child–Pugh A liver function and a higher rate of discontinuation due to progressive disease in patients who had Child–Pugh B liver function (Table 3).

There were 61 Grade 1–2 AEs and 19 Grade 3–4 AEs reported among the 81 patients with Child–Pugh A liver function, and 13 Grade 1–2 AEs and 3 Grade 3–4 AEs reported among the 26 patients with Child–Pugh B liver function (Supplementary Table S1).

### 3.3. Demographics and Outcomes by Etiology of Liver Disease

Of the 107 patients with uHCC who received STRIDE, 64 (59.8%) had a viral etiology (1/107 [0.9%] hepatitis B virus [HBV], 36/107 [33.6%] HCV, and 27/107 [25.2%] ALD + HCV), while 43 (40.2%) had non-viral etiologies (16/107 [15.0%] ALD, 25/107 [23.4%] MASH, and 2/107 [1.9%]) other) (Table 2). Patients with a viral etiology were slightly younger (median age 70.9 [IQR, 67.3–74.1] years vs. 75.4 [IQR, 69.5–77.2] years; *p* = 0.0011), were more likely to be current smokers (vs. non-smokers, *p* = 0.0891), and were less likely to have ascites at baseline (*p* = 0.0606) than patients with non-viral etiology. Additionally, although patients who were white comprised the majority overall, their proportion was lower in the viral etiology group compared with the non-viral etiology group, while there was a higher proportion of patients who were black in the viral etiology group. Otherwise, both groups had similar demographics, tumor characteristics, BCLC Stage, Child–Pugh Class, and prior treatments (Table 2).

Median OS was 10.5 (95% CI, 7.0–25.6) months in patients with viral etiology and 9.0 (95% CI, 4.6–16.0) months in patients with non-viral etiology (Figure 2C). There were 14/64 (21.9%) patients with viral etiology who were ongoing durvalumab treatment at the time of data extraction versus 5/43 (11.6%) patients with non-viral etiology (Table 3). There were higher rates of discontinuation due to AEs in patients with non-viral etiology and higher rates of discontinuation due to patient preference in patients with viral etiology (Table 3).

There were 49 Grade 1–2 AEs and 17 Grade 3–4 AEs reported among the 64 patients with viral etiology, and 25 Grade 1–2 AEs and 5 Grade 3–4 AEs reported among the 43 patients with non-viral etiology (Supplementary Table S1).

### 3.4. Demographics and Outcomes by Prior Receipt of Non-Systemic Therapies

Of the 107 patients with uHCC who received STRIDE, 77 (72.0%) had received prior non-systemic therapies, while 30 (28.0%) had received first-line systemic therapies. For those who had received prior non-systemic therapies, 8/77 (10.4%) underwent resection, 16/77 (20.8%) underwent ablation, 41/77 (53.2%) underwent transarterial embolization or transarterial chemoembolization (TAE/TACE), and 12/77 (15.6%) underwent transarterial radioembolization (TARE) as their first-line treatment (Table 4), with a median of 2.0 (IQR, 1.0–2.0) total LRTs among patients who received prior non-systemic therapies.

Patients who had not received prior non-systemic therapies were similar in age to those who had received prior non-systemic therapies (median age 71.0 [IQR, 67.0–75.9] vs. 72.6 [IQR, 68.5–76.3] years, *p* = 0.4455), with no statistical difference between groups regarding race/ethnicity, underlying liver disease etiology, baseline eCTP score, or modified ALBI grade (Table 2).

Those who had not received prior non-systemic therapies had a higher median largest tumor size compared with those who had received prior non-systemic therapies, (9.60 [IQR, 5.20–14.73] cm vs. 2.90 [IQR, 1.70–5.90] cm, *p* < 0.0001), larger median total tumor diameter (14.90 [IQR, 8.98–27.55] cm vs. 5.60 [IQR, 2.60–11.30] cm, *p* < 0.0001), and a higher rate of macrovascular invasion (17/30 [56.7%] vs. 23/77 [29.9%], *p* = 0.0187; Table 2). There was no significant difference in the presence of malignant lymph nodes, local invasion, or baseline alpha-fetoprotein (Table 2).

Median OS was 7.7 (95% CI, 2.8–17.3) months for patients who had not received prior non-systemic therapies and 11.1 (95% CI, 7.6–17.6) months for patients who had received prior non-systemic therapies (Figure 2D). For patients who had not received prior non-systemic therapies, there were 2/30 (6.7%) patients who were ongoing durvalumab treatment at the time of data extraction versus 17/77 (22.1%) patients who had received prior non-systemic therapies (Table 3). There were higher rates of discontinuation due to AEs among those who had received prior non-systemic therapies, while discontinuation due to progressive disease was more common among patients who had not received prior non-systemic therapies (Table 3).

There were60 Grade 1–2 AEs and 18 Grade 3–4 AEs reported among the 77 patients who had received prior non-systemic therapies, and 14 Grade 1–2 AEs and4 Grade 3–4 AEs reported among the 30 patients who had not received prior non-systemic therapies (Supplementary Table S1).

### 3.5. Exploration of Differential Outcomes for Patients Likely to Have Been Excluded from the HIMALAYA Study

The HIMALAYA study excluded patients with Child–Pugh B ≥ 7 cirrhosis, Vp4 portal vein thrombosis, severe ascites, or ECOG PS ≥ 2 [4]. Of the 107 patients, 37 patients would likely have been excluded from HIMALAYA, 3 patients due to ECOG PS ≥ 2, 20 patients due to Vp4 portal vein thrombosis, 11 patients due to Child–Pugh B liver function ≥ 7, and 1 patient due to severe ascites or related complications (Supplementary Table S2). Median OS (95% CI) was not unexpectedly shorter, 7.3 (3.4–9.6) months for likely excluded versus 12.6 (9.1–22.1) months among likely included patients, with a Cox proportional hazard ratio of 1.98 (95% CI, 1.26–3.14).

## 4. Discussion

Compared with the general US population, the VA population generally has an increased risk of HCC [18]. This analysis provides a unique opportunity to understand STRIDE as a treatment for uHCC in routine clinical practice and provides real-world data for STRIDE in patients with Child–Pugh B cirrhosis, while also assessing whether HCC etiology or prior receipt of non-systemic therapies affect outcomes in this population. In this real-world study of 107 patients in the VA healthcare system who received STRIDE as a first-line systemic therapy for uHCC, the incidence of Grade 3 or 4 AEs was low overall. Notably only 12% of all AEs were Grade 3–4 in patients with Child–Pugh B liver function, suggesting that STRIDE may be a suitable treatment option for this difficult-to-treat population. Patients with Child–Pugh B liver function are considered to have a higher risk of complications and lower benefit associated with treatments compared with patients with Child–Pugh A liver function [19], and are consequently excluded from many major clinical trials in uHCC, or their enrollment is limited [4,7,20,21]. Child–Pugh liver function is frequently missing from EMRs. For this reason, the methodology previously described by Kaplan et al. was used to assess Child–Pugh liver function in this study [17]. Based on this methodology, 24% of patients in this study had Child–Pugh B liver function, and 42% of these patients with Child–Pugh B liver function had Vp4 macrovascular invasion. In the US, many patients either present with or decline to Child–Pugh B liver function; therefore, there is a need to understand the efficacy and safety of HCC treatments in this population [9]. Retrospective studies have investigated ICI monotherapies, such as nivolumab monotherapy and durvalumab monotherapy, in patients who are not candidates for treatment with either STRIDE or atezolizumab plus bevacizumab [22,23]. Results from these studies showed a low incidence of Grade 3 or 4 AEs, suggesting that immunotherapies may be tolerated in patients with advanced HCC, including those with Child–Pugh B liver function [22,23]. However, in a retrospective study of patients with advanced HCC who received atezolizumab plus bevacizumab, incidence of Grade 3 or 4 AEs was significantly higher in the Child–Pugh B cohort compared with the Child–Pugh A cohort (48.1% vs. 17.7%, respectively) [24]. In contrast, in this analysis, there was no increase in Grade 3 or 4 AEs in patients with Child–Pugh B liver function compared to those with Child–Pugh A liver function, and no Grade 5 events in either group. The SIERRA study (NCT05883644) is assessing the safety and efficacy of STRIDE in a broader patient population than the HIMALAYA study, including participants with Child–Pugh B7/B8 liver function [25]. Early safety results from this study have shown a low incidence of Grade 3 or 4 AEs that were possibly related to study treatment [25].

In this analysis, OS outcomes were more favorable in patients who had Child–Pugh A versus Child–Pugh B liver function, but comparable to participants who met the inclusion criteria for the HIMALAYA study (Child–Pugh Class A, ECOG PS 0–1, no Vp4, and no prior systemic treatment for uHCC). While limited by the small sample size of the Child–Pugh B cohort (*n* = 26), these results are consistent with the expected, poorer prognosis associated with more impaired liver function, although noting that baseline liver function was maintained during treatment with STRIDE among survivors, consistent with observations from HIMALAYA. In a retrospective cohort study of patients in the VA healthcare system diagnosed with uHCC who initiated atezolizumab plus bevacizumab, sorafenib, or lenvatinib as a first-line treatment, a survival benefit was observed with atezolizumab plus bevacizumab compared with multikinase inhibitor comparators in patients with Child–Pugh A liver function, but no survival benefit was observed in patients with Child–Pugh B [26].

In this study, there was no significant difference in OS for those with viral versus non-viral etiologies. Previously, it has been hypothesized that those with HBV-associated HCC may have improved survival outcomes when treated with ICIs, though the true impact of etiology on efficacy remains controversial [10,27,28,29,30,31,32]. In the HIMALAYA study, STRIDE demonstrated a survival benefit compared with sorafenib irrespective of HCC etiology [12]. Findings from the present analysis provide further evidence that the survival benefit with STRIDE is observed irrespective of HCC etiology. Interestingly, in an analysis of patients in the VA healthcare system with uHCC treated with atezolizumab and bevacizumab, a median OS of 16.3 months versus 6.1 months was observed in patients with a viral versus non-viral etiology, respectively [26].

Approximately 72% of patients in this study received prior non-systemic therapies including resection, ablation, TAE/TACE, or TARE. Prior LRT, while an effective option for local disease control, may impair liver function, and is hypothesized to reduce the benefit of systemic therapy [13]. However, results from phase 3 clinical trials such as EMERALD-1 and LEAP-012 suggest that LRT combined with systemic therapy could lead to improved outcomes [33,34]. In this analysis, median OS was longer for those who received prior non-systemic therapies than those who did not (11.1 vs. 7.7 months). Likewise, in a previous retrospective study of patients in the VA healthcare system treated with atezolizumab plus bevacizumab, those who received prior LRT had numerically longer survival compared with those who had not received prior LRT [35]. This could be because patients who are eligible for LRT typically present with localized disease and, as such, may have better prognosis.

Clinical trials frequently enroll healthier, younger patient populations that are not fully representative of the real-world uHCC population, creating gaps in knowledge in older patients and those with more advanced disease [4,7,20,21]. Prospective trials investigating broader populations, such as the SIERRA uHCC trial, will inform clinical practice [25]. Additionally, studies of large, real-world data sets, like the present study, can serve as an alternative means of providing information to guide clinical practice.

In this real-world study, the patient population differed from the phase 3 HIMALAYA study, thus it was expected that efficacy and safety outcomes would differ between these studies and among the cohorts [4]. Although it is noteworthy that in the sensitivity analysis limited to patients meeting HIMALAYA inclusion criteria, the median OS was 12.6 (95% CI, 9.1–22.1) months, which is comparable to the OS observed in the HIMALAYA trial (16.43 [95% CI, 14.16–19.58] months). In general, the HIMALAYA study enrolled slightly younger participants (median age in the STRIDE arm was 65.0 [range, 22–86] years), extrahepatic spread was higher (53.2%), and overall macrovascular invasion was lower (26.2%) in the STRIDE arm of the HIMALAYA study compared with this VA cohort [4]. While the proportion of patients with non-viral etiology was similar between the HIMALAYA study (41% of participants in the STRIDE arm) and the present analysis (40% of patients in the present analysis), the HIMALAYA study only permitted participants with Child–Pugh A liver function; therefore, the prognosis in this VA cohort is expected to be poorer than the HIMALAYA study population [4]. The median OS observed with STRIDE in the present study is higher than historical comparators in this VA population (median OS 8.0 months with sorafenib) [26]. Our findings are further supported by a recent real-world analysis of participants with uHCC treated with durvalumab and/or tremelimumab which showed a median OS of 14.6 months; however, direct comparisons cannot be drawn between studies due to differences in the study population and conduct [36]. Overall, these findings suggest that STRIDE may be a suitable treatment option for patients who may have been excluded from the HIMALAYA study.

One of the strengths of this study was the use of a large national cohort of patients from the VA with diverse demographic information, allowing for detailed subgroup analyses. The use of comprehensive manual chart abstraction from the VA database ensured a quality review of the data. However, there are several limitations and potential sources of bias that accompany retrospective cohort studies. The VA population is predominantly male, typically older and may have more comorbidities than the broader US population [18]. While the enriched risk factors in the VA population allowed for this relatively large cohort, these differences may mean these results may not be generalizable to all uHCC populations. In this study, some patients in the VA system may have had more than one insurance plan and data on procedures conducted outside of the VA system would not be available for this analysis. Furthermore, data were limited to what was available in the EMR. In some EMRs, data, such as Child–Pugh liver function, may be missing, inaccurate, or inconsistent for some elements in the CDW/EMR. In addition, this analysis included a relatively short follow-up period for some patients (thus not unveiling the complete clinical benefit of STRIDE, which enables long-term OS outcomes [8]), and a small sample size in certain subgroups. Finally, as patients were not randomly assigned to STRIDE, this is a potential point of bias.

## 5. Conclusions

This real-world analysis investigated factors that can influence treatment response in HCC, including etiology, reduced liver function, and prior non-systemic therapies. Notably, STRIDE had a manageable safety profile in patients with Child–Pugh B liver function (often excluded from clinical studies), with no observed Grade 5 AEs and a relatively low incidence of Grade 3 or 4 AEs, consistent with an interim analysis of the SIERRA study. Additionally, there was preserved liver function in survivors on STRIDE and a median OS favorable to historical comparators [35]. Future studies in larger, more representative databases and additional prospective studies may further support these findings and inform treatment decision-making in this heterogenous patient population. Overall, these real-world data confirm the encouraging safety and efficacy of STRIDE among a broad range of patients with uHCC, reinforcing its position as a standard of care for first-line treatment. The study also suggests that there is the potential for individualization of treatment based on factors such as viral etiology, liver function, and prior non-systemic therapies to optimize patient outcomes.

## Figures and Tables

**Table 1:** Treatment information.

	Overall	Child– Pugh A	Child–Pugh B	Viral Etiology	Non-Viral Etiology	Prior Non-Systemic Therapy	No Prior Non-Systemic Therapy
Median time (days) from HCC diagnosis to STRIDE (IQR)	423.0 (151.5–1028.5)	518.0 (182.0–1274.0)	187.0 (43.5–733.8)	576.0 (165.8–1338.0)	336.0 (63.5–717.0)	685.0 (361.0–1377.0)	42.5 (28.5–65.8)
Median number of durvalumab cycles (IQR)	4.0 (2.0–8.0)	4.0 (2.0–9.0)	3.0 (1.0–4.0)	4.0 (2.0–9.0)	4.0 (2.0–6.0)	4.0 (2.0–8.0)	3.0 (1.3–5.8)
Median length (days) of follow-up (IQR)	287.0 (107.5–528.5)	373.0 (128.0–591.0)	155.0 (44.3–278.5)	315.5 (117.3–596.3)	269.0 (88.5–481.0)	333.0 (126.0–551.0)	231.5 (77.8–466.5)

Abbreviations: HCC, hepatocellular carcinoma; IQR, interquartile range; STRIDE, Single Tremelimumab Regular Interval Durvalumab.

**Table 2:** Patient demographics and disease characteristics.

	Overall	Child–Pugh		Viral Etiology		Prior Non-Systemic Therapies	
	Total (*n* = 107)	A (*n* = 81)	B (*n* = 26)	No (*n* = 43)	Yes (*n* = 64)	No (*n* = 30)	Yes (*n* = 77)
Median age, years (IQR)	72.2 (68.0–76.1)	72.1 (68.5–76.4)	72.9 (66.8–76.1)	75.4 (69.5–77.2)	70.9 (67.3–74.1)	71.0 (67.0–75.9)	72.6 (68.5–76.3)
Male, *n* (%)	107 (100.0)	81 (100.0)	26 (100.0)	43 (100.0)	64 (100.0)	30 (100.0)	77 (100.0)
Race/ethnicity, *n* (%)							
White	67 (62.6)	48 (59.3)	19 (73.1)	30 (69.8)	37 (57.8)	20 (66.7)	47 (61.0)
Black	19 (17.8)	16 (19.8)	3 (11.5)	3 (7.0)	16 (25.0)	8 (26.7)	11 (14.3)
Hispanic	13 (12.1)	9 (11.1)	4 (15.4)	8 (18.6)	5 (7.8)	1 (3.3)	12 (15.6)
Asian	1 (0.9)	1 (1.2)	0 (0.0)	1 (2.3)	0 (0.0)	0 (0.0)	1 (1.3)
Other	6 (5.6)	6 (7.4)	0 (0.0)	1 (2.3)	5 (7.8)	1 (3.3)	5 (6.5)
Tobacco use, *n* (%)							
Never	31 (29.0)	26 (32.1)	5 (19.2)	16 (37.2)	15 (23.4)	7 (23.3)	24 (31.2)
Former smoker	35 (32.7)	23 (28.4)	12 (46.2)	15 (34.9)	20 (31.3)	12 (40.0)	23 (29.9)
Current smoker	38 (35.5)	29 (35.8)	9 (34.6)	10 (23.3)	28 (43.8)	9 (30.0)	29 (37.7)
Median BMI, kg/m^2^ (IQR)	27.2 (23.6–31.6)	26.9 (23.5–30.4)	29.0 (24.6–34.6)	27.5 (26.0–31.5)	26.6 (22.5–31.5)	26.1 (21.9–29.8)	27.5 (24.9–31.9)
Etiology, *n* (%)							
ALD	16 (15.0)	7 (8.6)	9 (34.6)	16 (37.2)	0 (0.0)	6 (20.0)	10 (13.0)
HCV	36 (33.6)	32 (39.5)	4 (15.4)	0 (0.0)	36 (56.3)	11 (36.7)	25 (32.5)
ALD + HCV	27 (25.2)	21 (25.9)	6 (23.1)	0 (0.0)	27 (42.2)	5 (16.7)	22 (28.6)
MASH	25 (23.4)	18 (22.2)	7 (26.9)	25 (58.1)	0 (0.0)	7 (23.3)	18 (23.4)
HBV	1 (0.9)	1 (1.2)	0 (0.0)	0 (0.0)	1 (1.6)	0 (0.0)	1 (1.3)
Other	2 (1.9)	2 (2.5)	0 (0.0)	2 (4.7)	0 (0.0)	1 (3.3)	1 (1.3)
eCTP score, ^a^ *n* (%)							
5	48 (44.9)	48 (59.3)	0 (0.0)	15 (34.9)	33 (51.6)	8 (26.7)	40 (51.9)
6	33 (30.8)	33 (40.7)	0 (0.0)	12 (27.9)	21 (32.8)	11 (36.7)	22 (28.6)
7	15 (14.0)	0 (0.0)	15 (57.7)	8 (18.6)	7 (10.9)	4 (13.3)	11 (14.3)
8	9 (8.4)	0 (0.0)	9 (34.6)	6 (14.0)	3 (4.7)	6 (20.0)	3 (3.9)
9	2 (1.9)	0 (0.0)	2 (7.7)	2 (4.7)	0 (0.0)	1 (3.3)	1 (1.3)
mALBI grade, *n* (%)							
1	24 (22.4)	24 (29.6)	0 (0.0)	6 (14.0)	18 (28.1)	3 (10.0)	21 (27.3)
2A	20 (18.7)	20 (24.7)	0 (0.0)	9 (20.9)	11 (17.2)	4 (13.3)	16 (20.8)
2B	48 (44.9)	33 (40.7)	15 (57.7)	17 (39.5)	31 (48.4)	18 (60.0)	30 (39.0)
3	13 (12.1)	3 (3.7)	10 (38.5)	10 (23.3)	3 (4.7)	5 (16.7)	8 (10.4)
ECOG PS > 2 at start of systemic therapy, *n* (%)	3 (2.8)	2 (2.5)	1 (3.8)	1 (2.3)	2 (3.1)	0 (0.0)	3 (3.9)
BCLC Stage, *n* (%)							
A	7 (6.5)	4 (4.9)	3 (11.5)	4 (9.3)	3 (4.7)	2 (6.7)	5 (6.5)
B	33 (30.8)	29 (35.8)	4 (15.4)	18 (41.9)	15 (23.4)	5 (16.7)	28 (36.4)
C	66 (61.7)	47 (58.0)	19 (73.1)	21 (48.8)	45 (70.3)	23 (76.7)	43 (55.8)
D	1 (0.9)	1 (1.2)	0 (0.0)	0 (0.0)	1 (1.6)	0 (0.0)	1 (1.3)
Baseline ascites, *n* (%)							
0	3 (2.8)	3 (3.7)	0 (0.0)	2 (4.7)	1 (1.6)	2 (6.7)	1 (1.3)
1	95 (88.8)	76 (93.8)	19 (73.1)	34 (79.1)	61 (95.3)	24 (80.0)	71 (92.2)
2	8 (7.5)	2 (2.5)	6 (23.1)	6 (14.0)	2 (3.1)	4 (13.3)	4 (5.2)
3	1 (0.9)	0 (0.0)	1 (3.8)	1 (2.3)	0 (0.0)	0 (0.0)	1 (1.3)
Extrahepatic spread, *n* (%)	24 (22.4)	22 (27.2)	2 (7.7)	6 (14.0)	18 (28.1)	7 (23.3)	17 (22.1)
Macrovascular invasion, *n* (%)							
None	67 (62.6)	56 (69.1)	11 (42.3)	26 (60.5)	41 (64.1)	13 (43.3)	54 (70.1)
Any	40 (37.4)	25 (30.9)	15 (57.7)	17 (39.5)	23 (35.9)	17 (56.7)	23 (29.9)
Vp2	2 (1.9)	2 (2.5)	0 (0.0)	1 (2.3)	1 (1.6)	0 (0.0)	2 (2.6)
Vp3	13 (12.1)	11 (13.6)	2 (7.7)	5 (11.6)	8 (12.5)	4 (13.3)	9 (11.7)
Vp4	20 (18.7)	9 (11.1)	11 (42.3)	9 (20.9)	11 (17.2)	10 (33.3)	10 (13.0)
Not characterized	5 (4.7)	3 (3.7)	2 (7.7)	2 (4.7)	3 (4.7)	3 (10.0)	2 (2.6)
Median AFP, ng/mL (IQR)	110.2 (7.1–897.6)	60.2 (6.7–604.8)	473.4 (17.1–2067.1)	263.3 (38.1–821.5)	51.1 (6.6–1349.0)	228.7 (8.7–2067.1)	94.7 (6.8–643.4)
Median largest tumor, cm (IQR)	4.00 (2.10–8.70)	3.30 (2.00–8.50)	4.45 (2.55–10.80)	4.00 (2.30–8.85)	3.75 (2.15–8.40)	9.60 (5.20–14.73)	2.90 (1.70–5.90)
Median total tumor diameter, cm (IQR)	8.00 (3.70–16.40)	8.10 (3.50–18.70)	5.95 (4.53–14.95)	8.30 (5.05–14.65)	7.30 (3.30–18.95)	14.90 (8.98–27.55)	5.60 (2.60–11.30)
Malignant lymph nodes present, *n* (%)	31 (29.0)	27 (33.3)	4 (15.4)	9 (20.9)	22 (34.4)	9 (30.0)	22 (28.6)
Local invasion, *n* (%)	2 (1.9)	2 (2.5)	0 (0.0)	0 (0.0)	2 (3.1)	0 (0.0)	2 (2.6)
Number of intrahepatic tumors, *n* (%)							
None in liver	11 (10.3)	9 (11.1)	2 (7.7)	3 (7.0)	8 (12.5)	0 (0.0)	11 (14.3)
1	27 (25.2)	18 (22.2)	9 (34.6)	12 (27.9)	15 (23.4)	11 (36.7)	16 (20.8)
2	14 (13.1)	10 (12.3)	4 (15.4)	5 (11.6)	9 (14.1)	2 (6.7)	12 (15.6)
3	9 (8.4)	7 (8.6)	2 (7.7)	2 (4.7)	7 (10.9)	0 (0.0)	9 (11.7)
4	6 (5.6)	5 (6.2)	1 (3.8)	4 (9.3)	2 (3.1)	4 (13.3)	2 (2.6)
5	16 (15.0)	13 (16.0)	3 (11.5)	8 (18.6)	8 (12.5)	3 (10.0)	13 (16.9)
6	24 (22.4)	19 (23.5)	5 (19.2)	9 (20.9)	15 (23.4)	10 (33.3)	14 (18.2)
Infiltrative/innumerable	0 (0.0)	0 (0.0)	0 (0.0)	0 (0.0)	0 (0.0)	0 (0.0)	0 (0.0)

Percentages may not add to 100 due to missing data. ^a^ eCTP score were determined at the time of initiation of systemic therapy using the methods described in Kaplan et al. 2015 [17]. Abbreviations: AFP, alpha-fetoprotein; ALD, alcoholic liver disease; BCLC, Barcelona Clinic Liver Cancer; BMI, body mass index; ECOG PS, Eastern Cooperative Oncology Group performance status; eCTP, expanded Child–Turcotte–Pugh; HBV, hepatitis B virus; HCV, hepatitis C virus; mALBI, modified albumin–bilirubin; MASH, metabolic dysfunction-associated steatohepatitis; Vp2, vascular invasion in the portal vein (second degree); Vp3, vascular invasion in the portal vein (third degree); Vp4, vascular invasion in the portal vein (fourth degree).

**Table 3:** Exposure to STRIDE treatment.

	Overall	Child–Pugh		Viral Etiology		Prior Non-Systemic Therapies	
	Total (*n* = 107)	A (*n* = 81)	B (*n* = 26)	No (*n* = 43)	Yes (*n* = 64)	No (*n* = 30)	Yes (*n* = 77)
Median cycles of durvalumab received, (IQR)	4.0 (2.0–8.0)	4.0 (2.0–9.0)	3.0 (1.0–4.0)	4.0 (2.0–6.0)	4.0 (2.0–9.0)	3.0 (1.3–5.8)	4.0 (2.0–8.0)
Ongoing treatment at index, *n* (%)	19 (17.8)	19 (23.5)	0 (0.0)	5 (11.6)	14 (21.9)	2 (6.7)	17 (22.1)
Discontinuation reason, *n* (%)							
Death	1 (0.9)	1 (1.2)	0 (0.0)	0 (0.0)	1 (1.6)	0 (0.0)	1 (1.3)
Progressive disease	22 (20.6)	11 (13.6)	11 (42.3)	9 (20.9)	13 (20.3)	7 (23.3)	15 (19.5)
Adverse event	43 (40.2)	35 (43.2)	8 (30.8)	24 (55.8)	19 (29.7)	9 (30.0)	34 (44.2)
Patient preference	4 (3.7)	4 (4.9)	0 (0.0)	0 (0.0)	4 (6.3)	0 (0.0)	4 (5.2)
Other	9 (8.4)	7 (8.6)	2 (7.7)	2 (4.7)	7 (10.9)	7 (23.3)	2 (2.6)
Median length (days) of follow-up (IQR)	287.0 (107.5–528.5)	373.0 (128.0–591.0)	155.0 (44.3–278.5)	269.0 (88.5–481.0)	315.5 (117.3–596.3)	231.5 (77.8–466.5)	333.0 (126.0–551.0)
Death during follow-up, *n* (%)	77 (72.0)	52 (64.2)	25 (96.2)	36 (83.7)	41 (64.1)	24 (80.0)	53 (68.8)
Censored, *n* (%)	30 (28.0)	29 (35.8)	1 (3.8)	7 (16.3)	23 (35.9)	6 (20.0)	24 (31.2)

**Table 4:** Treatment for HCC prior to and following STRIDE.

	Overall	Child–Pugh		Viral Etiology		Prior Non-Systemic Therapies	
	Total (*n* = 107)	A (*n* = 81)	B (*n* = 26)	No (*n* = 43)	Yes (*n* = 64)	No (*n* = 30)	Yes (*n* = 77)
Initial treatment, n (%)							
Resection	8 (7.5)	8 (9.9)	0 (0.0)	3 (7.0)	5 (7.8)	0 (0.0)	8 (10.4)
Ablation	16 (15.0)	14 (17.3)	2 (7.7)	1 (2.3)	15 (23.4)	0 (0.0)	16 (20.8)
TAE/TACE	41 (38.3)	30 (37.0)	11 (42.3)	20 (46.5)	21 (32.8)	0 (0.0)	41 (53.2)
TARE	12 (11.2)	10 (12.3)	2 (7.7)	5 (11.6)	7 (10.9)	0 (0.0)	12 (15.6)
Systemic (STRIDE)	30 (28.0)	19 (23.5)	11 (42.3)	14 (32.6)	16 (25.0)	30 (100.0)	0 (0.0)
Received LRT as initial treatment, n (%)	69 (64.5)	54 (66.7)	15 (57.7)	26 (60.5)	43 (67.2)	0 (0.0)	69 (89.6)
Initial LRT type, n (%)							
None	30 (28.0)	19 (23.5)	11 (42.3)	14 (32.6)	16 (25.0)	30 (100.0)	0 (0.0)
Ablation	16 (15.0)	14 (17.3)	2 (7.7)	1 (2.3)	15 (23.4)	0 (0.0)	16 (20.8)
cTACE	6 (5.6)	3 (3.7)	3 (11.5)	2 (4.7)	4 (6.3)	0 (0.0)	6 (7.8)
TAE	1 (0.9)	1 (1.2)	0 (0.0)	0 (0.0)	1 (1.6)	0 (0.0)	1 (1.3)
DEB-TACE	34 (31.8)	26 (32.1)	8 (30.8)	18 (41.9)	16 (25.0)	0 (0.0)	34 (44.2)
Y90 TARE	12 (11.2)	10 (12.3)	2 (7.7)	5 (11.6)	7 (10.9)	0 (0.0)	12 (15.6)
Resection	2 (1.9)	2 (2.5)	0 (0.0)	0 (0.0)	2 (3.1)	0 (0.0)	2 (2.6)
Received additional LRTs, n (%)	44 (44.1)	35 (43.2)	9 (34.6)	15 (34.9)	29 (45.3)	0 (0.0)	44 (57.1)
Median time from STRIDE to next LRT, days (IQR)	120.0 (33.0–145.0)	120.5 (54.8–139.0)	120.0 (76.5–156.5)	33.0 (33.0–120.0)	169.0 (139.0–193.0)	120.0 (76.5–132.5)	120.5 (54.8–175.0)
Treatments received after STRIDE, n (%)							
Resection	0 (0.0)	0 (0.0)	0 (0.0)	0 (0.0)	0 (0.0)	0 (0.0)	0 (0.0)
Ablation	0 (0.0)	0 (0.0)	0 (0.0)	0 (0.0)	0 (0.0)	0 (0.0)	0 (0.0)
TAE/TACE	1 (0.9)	1 (1.2)	0 (0.0)	1 (2.3)	0 (0.0)	0 (0.0)	1 (1.3)
TARE	1 (0.9)	1 (1.2)	0 (0.0)	0 (0.0)	1 (1.6)	0 (0.0)	1 (1.3)
Radiation	0 (0.0)	0 (0.0)	0 (0.0)	0 (0.0)	0 (0.0)	0 (0.0)	0 (0.0)
Sorafenib	1 (0.9)	1 (1.2)	0 (0.0)	0 (0.0)	1 (1.6)	0 (0.0)	1 (1.3)
Lenvatinib	2 (1.9)	1 (1.2)	1 (3.8)	2 (4.7)	0 (0.0)	0 (0.0)	2 (2.6)
Nivolumab	0 (0.0)	0 (0.0)	0 (0.0)	0 (0.0)	0 (0.0)	0 (0.0)	0 (0.0)
Pembrolizumab	0 (0.0)	0 (0.0)	0 (0.0)	0 (0.0)	0 (0.0)	0 (0.0)	0 (0.0)
Atezolizumab/bevacizumab	0 (0.0)	0 (0.0)	0 (0.0)	0 (0.0)	0 (0.0)	0 (0.0)	0 (0.0)

Abbreviations: cTACE, conventional transarterial chemoembolization; DEB-TACE, drug-eluting bead transarterial chemoembolization; LRT, locoregional therapy; TACE, transarterial chemoembolization; TAE, transarterial embolization; TARE, transarterial radioembolization; Y90 TARE, Yttrium-90 transarterial radioembolization.

## Data Availability

Results do not reflect the views of the U.S. Government. Data underlying the findings described in this manuscript may be obtained in accordance with AstraZeneca’s data sharing policy described at https://www.astrazenecaclinicaltrials.com/our-transparency-commitments/. Individual patient data can be shared upon request under the terms of DUA/DTA with domestic parties only.

## Supplementary Materials

The following supporting information can be downloaded at: https://www.mdpi.com/article/10.3390/cancers18071085/s1, Table S1. Incidence of adverse events by Common Terminology Criteria for Adverse Events (CTCAE) Grade. Table S2. Patient demographics and disease characteristics for participants likely to have been included or excluded from the HIMALAYA study.
