# Supplementary material for: Outcomes with Single Tremelimumab Regular Interval Durvalumab (STRIDE) for Unresectable Hepatocellular Carcinoma in the US Veterans Administration
Source: Cancers (Basel). 2026 Mar 26;18(7):1085. doi: 10.3390/cancers18071085 (PMC13072021; doi:10.3390/cancers18071085)
Supplement: Supplementary file 1 [file cancers-18-01085-s001.zip › cancers-4167154-supplementary.pdf]

Outcomes with Single Tremelimumab Regular Interval Durvalumab (STRIDE) for Unresectable Hepatocellular Carcinoma in the US Veterans Administration

Shalini Bansal[1,2], Priya Amin[3], Courtney Williamson[1], Stephen J Valerio[3], David E. Kaplan[1,4]

[1]*Corporal Michael J. Crescenz VA Medical Center, Gastroenterology Section, Philadelphia, PA, USA*

[2]*Thomas Jefferson University School of Medicine, Philadelphia, PA, USA*

[3]*AstraZeneca, Gaithersburg, MD, USA*

[4]*Perelman School of Medicine, University of Pennsylvania, Department of Medicine, Philadelphia, PA, USA*

Supplementary Information

Supplementary Table S1. Incidence of adverse events by Common Terminology Criteria for Adverse Events (CTCAE) Grade.

		Overall	Child-Pugh		Viral Etiology		Prior Non-Systemic Therapies	
	CTCAE Grade	Total (N = 107)	A (N = 81)	B (N = 26)	No (N = 43)	Yes (N = 64)	No (N = 30)	Yes (N = 77)
Abdominal pain	1–2	2 (1.9)	2 (2.5)	0 (0.0)	0 (0.0)	2 (3.1)	0 (0.0)	2 (2.6)
	3–4	0 (0.0)	0 (0.0)	0 (0.0)	0 (0.0)	0 (0.0)	0 (0.0)	0 (0.0)
Autoimmune hepatitis	1–2	1 (0.9)	1 (1.2)	0 (0.0)	1 (2.3)	0 (0.0)	1 (3.3)	0 (0.0)
	3–4	4 (3.7)	4 (4.9)	0 (0.0)	1 (2.3)	3 (4.7)	1 (3.3)	3 (3.9)
Colitis	1–2	0 (0.0)	0 (0.0)	0 (0.0)	0 (0.0)	0 (0.0)	0 (0.0)	0 (0.0)
	3–4	2 (1.9)	2 (2.5)	0 (0.0)	0 (0.0)	2 (3.1)	1 (3.3)	1 (1.3)
Confusion	1–2	1 (0.9)	0 (0.0)	1 (3.8)	0 (0.0)	1 (1.6)	0 (0.0)	1 (1.3)
	3–4	0 (0.0)	0 (0.0)	0 (0.0)	0 (0.0)	0 (0.0)	0 (0.0)	0 (0.0)
Cough	1–2	2 (1.9)	1 (1.2)	1 (3.8)	0 (0.0)	2 (3.1)	1 (3.3)	1 (1.3)
	3–4	0 (0.0)	0 (0.0)	0 (0.0)	0 (0.0)	0 (0.0)	0 (0.0)	0 (0.0)
Decreased appetite	1–2	3 (2.8)	3 (3.7)	0 (0.0)	0 (0.0)	3 (4.7)	2 (6.7)	1 (1.3)

	3–4	0 (0.0)	0 (0.0)	0 (0.0)	0 (0.0)	0 (0.0)	0 (0.0)	0 (0.0)
Diarrhea	1–2	9 (8.4)	6 (7.4)	3 (11.5)	4 (9.3)	5 (7.8)	2 (6.7)	7 (9.1)
	3–4	3 (2.8)	3 (3.7)	0 (0.0)	2 (4.7)	1 (1.6)	1 (3.3)	2 (2.6)
Dyspnea	1–2	3 (2.8)	2 (2.5)	1 (3.8)	1 (2.3)	2 (3.1)	0 (0.0)	3 (3.9)
	3–4	0 (0.0)	0 (0.0)	0 (0.0)	0 (0.0)	0 (0.0)	0 (0.0)	0 (0.0)
Elevated alanine aminotransferase	1–2	7 (6.5)	7 (8.6)	0 (0.0)	3 (7.0)	4 (6.3)	1 (3.3)	6 (7.8)
	3–4	0 (0.0)	0 (0.0)	0 (0.0)	0 (0.0)	0 (0.0)	0 (0.0)	0 (0.0)
Elevated aspartate aminotransferase	1–2	7 (6.5)	7 (8.6)	0 (0.0)	3 (7.0)	4 (6.3)	1 (3.3)	6 (7.8)
	3–4	0 (0.0)	0 (0.0)	0 (0.0)	0 (0.0)	0 (0.0)	0 (0.0)	0 (0.0)
Elevated bilirubin	1–2	1 (0.9)	1 (1.2)	0 (0.0)	0 (0.0)	1 (1.6)	0 (0.0)	1 (1.3)
	3–4	0 (0.0)	0 (0.0)	0 (0.0)	0 (0.0)	0 (0.0)	0 (0.0)	0 (0.0)
Elevated creatinine	1–2	1 (0.9)	0 (0.0)	1 (3.8)	0 (0.0)	1 (1.6)	0 (0.0)	1 (1.3)
	3–4	0 (0.0)	0 (0.0)	0 (0.0)	0 (0.0)	0 (0.0)	0 (0.0)	0 (0.0)
Fatigue	1–2	13 (12.1)	12 (14.8)	1 (3.8)	4 (9.3)	9 (14.1)	3 (10.0)	10 (13.0)
	3–4	1 (0.9)	1 (1.2)	0 (0.0)	0 (0.0)	1 (1.6)	0 (0.0)	1 (1.3)
Gastrointestinal bleed	1–2	0 (0.0)	0 (0.0)	0 (0.0)	0 (0.0)	0 (0.0)	0 (0.0)	0 (0.0)

	3–4	1 (0.9)	0 (0.0)	1 (3.8)	1 (2.3)	0 (0.0)	0 (0.0)	1 (1.3)
Hyperkalemia	1–2	0 (0.0)	0 (0.0)	0 (0.0)	0 (0.0)	0 (0.0)	0 (0.0)	0 (0.0)
	3–4	1 (0.9)	1 (1.2)	0 (0.0)	0 (0.0)	1 (1.6)	0 (0.0)	1 (1.3)
Hypertension	1–2	1 (0.9)	1 (1.2)	0 (0.0)	0 (0.0)	1 (1.6)	0 (0.0)	1 (1.3)
	3–4	1 (0.9)	1 (1.2)	0 (0.0)	0 (0.0)	1 (1.6)	0 (0.0)	1 (1.3)
Hyponatremia	1–2	0 (0.0)	0 (0.0)	0 (0.0)	0 (0.0)	0 (0.0)	0 (0.0)	0 (0.0)
	3–4	1 (0.9)	1 (1.2)	0 (0.0)	0 (0.0)	1 (1.6)	0 (0.0)	1 (1.3)
Hypothyroidism	1–2	1 (0.9)	1 (1.2)	0 (0.0)	1 (2.3)	0 (0.0)	0 (0.0)	1 (1.3)
	3–4	1 (0.9)	0 (0.0)	1 (3.8)	0 (0.0)	1 (1.6)	1 (3.3)	0 (0.0)
Muscle weakness	1–2	1 (0.9)	1 (1.2)	0 (0.0)	0 (0.0)	1 (1.6)	0 (0.0)	1 (1.3)
	3–4	0 (0.0)	0 (0.0)	0 (0.0)	0 (0.0)	0 (0.0)	0 (0.0)	0 (0.0)
Myalgias	1–2	1 (0.9)	0 (0.0)	1 (3.8)	0 (0.0)	1 (1.6)	0 (0.0)	1 (1.3)
	3–4	1 (0.9)	1 (1.2)	0 (0.0)	0 (0.0)	1 (1.6)	0 (0.0)	1 (1.3)
Myositis	1–2	0 (0.0)	0 (0.0)	0 (0.0)	0 (0.0)	0 (0.0)	0 (0.0)	0 (0.0)
	3–4	1 (0.9)	1 (1.2)	0 (0.0)	0 (0.0)	1 (1.6)	0 (0.0)	1 (1.3)
Nausea	1–2	4 (3.7)	3 (3.7)	1 (3.8)	0 (0.0)	4 (6.3)	0 (0.0)	4 (5.2)
	3–4	0 (0.0)	0 (0.0)	0 (0.0)	0 (0.0)	0 (0.0)	0 (0.0)	0 (0.0)
Peripheral edema	1–2	1 (0.9)	1 (1.2)	0 (0.0)	1 (2.3)	0 (0.0)	0 (0.0)	1 (1.3)

	3–4	0 (0.0)	0 (0.0)	0 (0.0)	0 (0.0)	0 (0.0)	0 (0.0)	0 (0.0)
Peripheral neuropathy	1–2	1 (0.9)	1 (1.2)	0 (0.0)	0 (0.0)	1 (1.6)	0 (0.0)	1 (1.3)
	3–4	0 (0.0)	0 (0.0)	0 (0.0)	0 (0.0)	0 (0.0)	0 (0.0)	0 (0.0)
Pneumonitis	1–2	0 (0.0)	0 (0.0)	0 (0.0)	0 (0.0)	0 (0.0)	0 (0.0)	0 (0.0)
	3–4	1 (0.9)	1 (1.2)	0 (0.0)	0 (0.0)	1 (1.6)	0 (0.0)	1 (1.3)
Rash	1–2	14 (13.1)	11 (13.6)	3 (11.5)	7 (16.3)	7 (10.9)	3 (10.0)	11 (14.3)
	3–4	0 (0.0)	0 (0.0)	0 (0.0)	0 (0.0)	0 (0.0)	0 (0.0)	0 (0.0)
Thyroiditis	1–2	0 (0.0)	0 (0.0)	0 (0.0)	0 (0.0)	0 (0.0)	0 (0.0)	0 (0.0)
	3–4	1 (0.9)	0 (0.0)	1 (3.8)	0 (0.0)	1 (1.6)	0 (0.0)	1 (1.3)
Type I diabetes mellitus	1–2	0 (0.0)	0 (0.0)	0 (0.0)	0 (0.0)	0 (0.0)	0 (0.0)	0 (0.0)
	3–4	2 (1.9)	2 (2.5)	0 (0.0)	1 (2.3)	1 (1.6)	0 (0.0)	2 (2.6)
Weight loss	1–2	0 (0.0)	0 (0.0)	0 (0.0)	0 (0.0)	0 (0.0)	0 (0.0)	0 (0.0)
	3–4	1 (0.9)	1 (1.2)	0 (0.0)	0 (0.0)	1 (1.6)	0 (0.0)	1 (1.3)

Data are presented as n (%).

Adverse events are sorted in alphabetical order by reported term.

CTCAE Grade 1: mild; Grade 2: moderate; Grade 3: severe; Grade 4: life-threatening. No Grade 5 adverse events were reported.

Supplementary Table S2. Patient demographics and disease characteristics for participants likely to have been included or excluded from the HIMALAYA study.

	Overall	Participants likely to have been included from the HIMALAYA study	
	**Total (N = 107)**	**No (N = 37)**	**Yes (N = 70)**
Median age, years (IQR)	72.2 (68.0–76.1)	70.9 (66.8–76.1)	72.7 (68.8–76.4)
Male, n (%)	107 (100.0)	37 (100.0)	70 (100.0)
Race/ethnicity, n (%)			
White	67 (62.6)	25 (67.6)	42 (60.0)
Black	19 (17.8)	4 (10.8)	15 (21.4)
Hispanic	13 (12.1)	6 (16.2)	7 (10.0)
Asian	1 (0.9)	0 (0.0)	1 (1.4)
Other	6 (5.6)	2 (5.4)	4 (5.7)
Tobacco use, n (%)			
Never	31 (29.0)	8 (21.6)	23 (32.9)
Former smoker	35 (32.7)	13 (35.1)	22 (31.4)
Current smoker	38 (35.5)	16 (43.2)	22 (31.4)

Median BMI, kg/m^2 (IQR)	27.2 (23.6–31.6)	28.2 (23.0–32.3)	27.1 (23.9–31.0)
Etiology, n (%)			
ALD	16 (15.0)	9 (24.3)	7 (10.0)
HCV	36 (33.6)	12 (32.4)	24 (34.3)
ALD+HCV	27 (25.2)	6 (16.2)	21 (30.0)
MASH	25 (23.4)	9 (24.3)	16 (22.9)
HBV	1 (0.9)	0 (0.0)	1 (1.4)
Other	2 (1.9)	1 (2.7)	1 (1.4)
eCTP score,[a] n (%)			
5	48 (44.9)	6 (16.2)	42 (60.0)
6	33 (30.8)	5 (13.5)	28 (40.0)
7	15 (14.0)	15 (40.5)	0 (0.0)
8	9 (8.4)	9 (24.3)	0 (0.0)
9	2 (1.9)	2 (5.4)	0 (0.0)
mALBI grade, n (%)			
1	24 (22.4)	3 (8.1)	21 (30.0)
2A	20 (18.7)	1 (2.7)	19 (27.1)
2B	48 (44.9)	20 (54.1)	28 (40.0)

3	13 (12.1)	11 (29.7)	2 (2.9)
ECOG PS ⩾2 at start of systemic therapy, n (%)	3 (2.8)	3 (8.1)	0 (0.0)
BCLC Stage, n (%)			
A	7 (6.5)	3 (8.1)	4 (5.7)
B	33 (30.8)	5 (13.5)	28 (40.0)
C	66 (61.7)	29 (78.4)	37 (52.9)
D	1 (0.9)	0 (0.0)	1 (1.4)
Baseline ascites, n (%)			
0	3 (2.8)	1 (2.7)	2 (2.9)
1	95 (88.8)	29 (78.4)	66 (94.3)
2	8 (7.5)	6 (16.2)	2 (2.9)
3	1 (0.9)	1 (2.7)	0 (0.0)
Extrahepatic spread, n (%)	24 (22.4)	4 (10.8)	20 (28.6)
Macrovascular invasion, n (%)			
None	67 (62.6)	13 (35.1)	54 (77.1)
Any	40 (37.4)	24 (64.9)	16 (22.9)
Vp2	2 (1.9)	0 (0.0)	2 (2.9)
Vp3	13 (12.1)	2 (5.4)	11 (15.7)

Vp4	20 (18.7)	20 (54.1)	0 (0.0)
Not characterized	5 (4.7)	2 (5.4)	3 (4.3)
Median AFP, ng/mL (IQR)	110.2 (7.1–897.6)	232.7 (7.8–1653.0)	94.2 (7.1–654.1)
Median largest tumor, cm (IQR)	4.00 (2.10–8.70)	4.10 (2.00–9.00)	3.40 (2.22–8.45)
Median total tumor diameter, cm (IQR)	8.00 (3.70–16.40)	5.80 (2.90–16.10)	8.15 (3.95–16.65)
Malignant lymph nodes present, n (%)	31 (29.0)	7 (18.9)	24 (34.3)
Local invasion, n (%)	2 (1.9)	0 (0.0)	2 (2.9)
Number of intrahepatic tumors, n (%)			
None in liver	11 (10.3)	4 (10.8)	7 (10.0)
1	27 (25.2)	12 (32.4)	15 (21.4)
2	14 (13.1)	4 (10.8)	10 (14.3)
3	9 (8.4)	3 (8.1)	6 (8.6)
4	6 (5.6)	1 (2.7)	5 (7.1)
5	16 (15.0)	7 (18.9)	9 (12.9)
6	24 (22.4)	6 (16.2)	18 (25.7)
Infiltrative/innumerable	0 (0.0)	0 (0.0)	0 (0.0)
Median OS, months (95% CI)	9.6 (7.3–12.6)	7.3 (3.4–9.6)	12.6 (9.1–22.1)

[a]eCTP score was determined at the time of initiation of systemic therapy using the methods described in Kaplan et al 2015 [1].

Abbreviations: AFP, alpha-fetoprotein; ALD, alcoholic liver disease; BCLC, Barcelona Clinic Liver Cancer; BMI, body mass index; CI, confidence interval; ECOG PS, Eastern Cooperative Oncology

Group performance status; eCTP, expanded Child-Turcotte-Pugh; HBV, hepatitis B virus; HCV, hepatitis C virus; IQR, interquartile ratio; mALBI, modified albumin-bilirubin; MASH, metabolic dysfunction-associated steatohepatitis; OS, overall survival; Vp2, vascular invasion in the portal vein (second degree); Vp3, vascular invasion in the portal vein (third degree); Vp4, vascular invasion in the portal vein (fourth degree).

References:

1. Kaplan, D.E.; Dai, F.; Aytaman, A.; Baytarian, M.; Fox, R.; Hunt, K.; Knott, A.; Pedrosa, M.; Pocha, C.; Mehta, R.; et al. Development and performance of an algorithm to estimate the Child-Turcotte-Pugh score from a national electronic healthcare database. *Clin Gastroenterol Hepatol* **2015**, *13*, 2333-41.e1-6. https://doi.org/10.1016/j.cgh.2015.07.010.
